# Youth Perceptions of Vaccination for COVID-19 in the United States

**DOI:** 10.1001/jamahealthforum.2021.2103

**Published:** 2021-08-20

**Authors:** Stephen M. Gorga, Eric J. Brandt, Julia Rosenberg, Marika E. Waselewski, Xochitl Amaro, Tammy Chang

**Affiliations:** 1 Institute for Healthcare Policy and Innovation, University of Michigan, Ann Arbor; 2 Division of Pediatric Critical Care Medicine, Department of Pediatrics, University of Michigan, Ann Arbor; 3 Division of Cardiovascular Medicine, Department of Internal Medicine, University of Michigan, Ann Arbor; 4 Section of General Pediatrics, Department of Pediatrics, Yale University School of Medicine, New Haven, Connecticut; 5 Department of Family Medicine, University of Michigan, Ann Arbor; 6 University of Michigan, Ann Arbor

## Abstract

This survey study examines opinions from a diverse sample of US youth after the initiation of mass immunization campaigns regarding COVID-19 vaccine acceptability, perceived barriers to vaccination, and anticipated changes in behavior.

## Introduction

Recent increases in COVID-19 cases are associated to infections among a younger population in the US.^1^ In a focused survey administered prior to vaccine emergency use authorizations (EUAs), 2 in 5 US youths reported willingness to vaccinate against COVID-19, whereas 1 in 3 were uncertain.^2^ Since then, the US has implemented a mass immunization campaign.^3^ Widespread vaccine uptake among youth is essential to achieve adequate community immunization levels to attenuate the COVID-19 pandemic.^4^ We therefore collected the thoughts and opinions from a diverse sample of US youth after the initiation of mass immunization campaigns regarding COVID-19 vaccine acceptability, perceived barriers to vaccination, and anticipated changes in behavior.

## Methods

Respondents were part of the MyVoice Poll of Youth, a national text message survey that collects the perspectives of youth (aged 14-24 years) on health and policy issues.^5^ Youths are recruited on a rolling basis to match national demographic benchmarks, including self-reported age, sex, race and ethnicity, education, and region of the country, based on weighted samples from the American Community Survey. This study was approved by the University of Michigan institutional review board, including a waiver of parental consent for minor participants. This study followed the American Association for Public Opinion Research (AAPOR) reporting guidelines. Online consent was obtained from all participants. Five open-ended questions were fielded on March 12, 2021, via text message regarding COVID-19 vaccination.

The authors developed a codebook through qualitative thematic analysis of responses. Responses were independently coded by 2 investigators (S.M.G., E.J.B., J.R., M.W., X.A.) using discussion to reach consensus. When appropriate, close-ended questions were first categorized (eg, yes, no) and the associated open-ended questions (“Why or why not?”) were then coded using thematic analysis to provide additional insight into these questions. Summary statistics of demographic data and code frequencies were calculated. Analyses were completed using Stata statistical software (version 16; StataCorp, LLC).

## Results

Of 1155 participants, 1074 responded to at least 1 question (response rate, 93%). The demographic characteristics of these respondents are shown in Table 1. In brief, respondents most commonly identified as male (n = 526 [49.0%]), and non-Hispanic–White individuals (n = 661 [61.7%]), with a mean (SD) age of 19.3 (2.4) years.

**Table 1.** Demographic Characteristics of the Full Cohort and the Vaccine-Willing Sample

Characteristic	No. (%)	
	Full cohort (n = 1074)	Vaccine willing (n = 797)
Age, mean (SD), y	19.2 (2.4)	19.4 (2.5)
14-15	47 (4.3)	33 (4.1)
16-24	1027 (95.6)	764 (95.9)
Gender identity		
Female	432 (40.2)	311 (39.0)
Male	526 (49.0)	389 (48.8)
Other^a^	116 (10.8)	97 (12.2)
Race and ethnicity		
No.	1072^b^	796^b^
Non-Hispanic White	661 (61.7)	500 (62.8)
Non-Hispanic Black	79 (7.4)	40 (5.0)
Non-Hispanic Asian	142 (13.2)	122 (15.3)
Hispanic	121 (11.3)	83 (10.4)
Non-Hispanic other^c^	69 (6.4)	51 (6.4)
Education		
High school or less	456 (42.5)	311 (39.0)
Some college or tech school	427 (39.8)	324 (40.7)
College or tech graduate	191 (17.8)	162 (20.3)
Region		
No.	1062^b^	788^b^
Midwest	343 (32.3)	252 (32.0)
South	291 (27.4)	209 (26.5)
West	229 (21.6)	181 (23.0)
Northeast	199 (18.7)	146 (18.5)
Locality		
No.	1063^b^	790^b^
Urban	365 (34.3)	270 (34.2)
Suburban	593 (55.8)	459 (77.4)
Rural	105 (9.9)	61 (7.7)

^a^ Other included the prespecified choices “nonbinary,” “transgender male,” “transgender female,” as well as “other,” in which the respondent could state how they identify.

^b^ Some participants did not provide all demographics.

^c^ Options included American Indian or Alaskan Native, Native Hawaiian or other Pacific Islander, and other (with self-description as they self-identify).

Table 2 summarizes the major themes, with representative quotes by question. Overall 797 of 1068 youth respondents (74.6%) were interested in getting vaccinated to protect themselves and return to normal (“Yes I will because I want to help stop the spread as well as get back to normal as soon as possible”). Of 1009 youth respondents, most were concerned about adverse effects (422 [41.8%]) and the effectiveness if the vaccine (118 [11.7%]), whereas 324 (32.1%) had no concerns. Of 990 youth respondents, 721 (72.8%) believed the vaccines are safe and/or effective, citing their trust in science (145 [20.1%]) and data (221 [30.7%]). To facilitate vaccination, youths indicated that they seek an easy sign-up process (“A simple streamlined process that’s centralized”) and locations close to them. Most youth also reported that they will continue mitigating behaviors such as wearing a mask even after vaccination.

**Table 2.** Questions, Themes, Responses, and Representative Quotes

Question/theme	No. (%)^a^	Representative quote
When a COVID-19 vaccine becomes available to you, will you get it? Why or why not? (n = 1068)		
Yes (includes already received)	797 (74.6)	
Protect self	279 (35.0)	“I work in a hospital so it’s important for me to protect myself…”
Normalcy	200 (25.1)	“Yes! I want to go back to normal life!”
Protect others	184 (23.1)	“I'll take it because then I can protect my family and community”
No	173 (16.2)	
Adverse effects	45 (26.0)	“No, because it has a lot of side effects”
Rushed	36 (20.8)	“No because it's not well tested yet.”
Does not trust the vaccine	27 (15.6)	“No. I don’t trust it.”
Uncertain	90 (8.4)	
Safety concerns	40 (44.4)	“I'm not sure. I'm afraid of the possible side effects.”
What concerns, if any, do you have about getting a COVID-19 vaccine? (n = 1009)		
None	324 (32.1)	“None, if the science is good then so am I”
Adverse effects	422 (41.8)	“…the commonly known and explicitly mentioned side effects, as well as the aforementioned possible unknown negative long term side effects.”
Not effective	118 (11.7)	“I am concerned that it will be less effective when more variants emerge”
Safety	84 (8.3)	“That it's unsafe in some way”
Rushed	56 (5.6)	“I just feel the vaccine is not that well developed yet”
Availability	52 (5.2)	“Maybe the vaccine will be delayed to my area”
Do you think that COVID-19 vaccines are safe and effective? Why or why not? (n = 990)		
Yes	721 (72.8)	“I think they are safe, yes”; “Yes, they are something like 93% effective”
Data and testing	221 (30.7)	“I think so, trials have shown that they are”
Trust the science	145 (20.1)	“Safe because I believe in science”
Trust the government	95 (13.2)	“Yes, I trust the govt organizations that approved it”
No	104 (10.5)	“I don't think it's safe bc of possible later on effects”; “Not really. A new strain of covid has just been discovered”
Not well tested	26 (25.0)	“No I don't think they have been through enough trials”
Too new	24 (23.1)	“No I don't trust how they just made it out of no where”
People hurt	19 (18.3)	“No. More people died from the vaccine than from plain covid”
Depends	90 (9.1)	“Possibly? I think they work but I'm not sure how safe they are”
What would make it easier for you to get vaccinated once you are eligible? (n = 930)		
More information	350 (37.6)	
Sign-up	159 (45.4)	“A clean online appointment scheduling tool”
Data	114 (32.6)	“More support and research that it's safe”
Locations	50 (14.3)	“Knowing where to get vaccinated”
Eligibility	35 (10.0)	“Definite lists of people that can get them and a National standard for who gets it next”
More access	406 (43.7)	
Proximity	199 (49.0)	“Having a vaccine center close to home”
Times	70 (17.2)	“more available appoin[t]ments”
Cost	66 (16.3)	“Make it free to me”
Transportation	42 (10.3)	“a ride to the vaccination center; i can't drive”
Nothing	75 (8.1)	“I can't think of anything, as soon as I'm eligible, I'll be getting the vaccine”
Unsure	65 (7.0)	
If you were to get vaccinated, would it change your behaviors/habits? How? (n = 952)		
Yes	370 (38.9)	
More travel/going out	161 (43.5)	“I think I would feel a bit safer when going out in public and I would maybe begin going to restaurants again...”
Seeing friends or family	121 (32.7)	“I would feel more comfortable doing things and seeing people”
Stop mitigating	55 (14.9)	“I think I could relax a little bit more about the COVID restrictions”
Seeing vaccinated people	50 (13.5)	“…I may stop wearing a mask around other vaccinated people as per the cdc guidelines”
No	537 (56.4)	
Continue mitigating	246 (45.8)	“I would feel more comfortable but wouldn't stop masking and social distancing”
Conditional	37 (6.9)	“I would still have the same habits for a while, at least until more of my circle gets vaccinated”
Not getting it	16 (3.0)	“No because I'm not getting vaccinated”
Uncertain	30 (3.2)	

^a^ Category percentages may not add up to 100 because codes are not mutually exclusive and only major codes are represented.

## Discussion

Whether or not to vaccinate against COVID-19 is an important health care decision many parents will soon make for their children and young adults must make for themselves. These findings indicate that youth respondents in this large, diverse sample were interested in receiving a COVID-19 vaccination. Our findings are limited to self-reported perceptions at the time of survey and do not necessarily predict future behavior. As younger demographic groups become eligible for vaccination,^6^ vaccination sites must be located in places that are convenient for youth and families, reduce complexity around making appointments, and must be prepared to administer vaccines to children.

Despite widespread trust in science and data, and a desire to return to normalcy, youth reported being concerned about short- and long-term adverse effects. Campaigns and educational programs can emphasize the safe and effective vaccination of millions of recipients in the US thus far and the integral role that widespread vaccination plays in returning to their usual activities.

## References

[1] United States Centers for Disease Control and Prevention. COVID Data Tracker Weekly Review. Accessed April 29, 2021. https://www.cdc.gov/coronavirus/2019-ncov/covid-data/covidview/index.html

[2] Brandt EJ, Rosenberg J, Waselewski ME, Amaro X, Wasag J, Chang T. National Study of Youth Opinions on Vaccination for COVID-19 in the U.S. J Adolesc Health. 2021;68(5):869-872. doi:10.1016/j.jadohealth.2021.02.013 33824070 PMC8019352

[3] United States Centers for Disease Control and Prevention. Guidelines for Mass COVID-19 Vaccination. Accessed April 29, 2021. https://www.cdc.gov/vaccines/covid-19/index.html

[4] Mahase E. Covid-19: Expedite vaccination or deaths will surge, researchers warn. BMJ. 2020;371:m4958. doi:10.1136/bmj.m4958 33376087

[5] DeJonckheere M, Nichols LP, Moniz MH, . MyVoice National Text Message Survey of Youth Aged 14 to 24 Years: Study Protocol. JMIR Res Protoc. 2017;6(12):e247. doi:10.2196/resprot.8502 29229587 PMC5742661

[6] The White House COVID-19 Response Team. Comments by Andrew Slavitt, Senior Advisor to the COVID-19 Response Coordinator. Accessed April 29, 2021. https://www.whitehouse.gov/briefing-room/press-briefings/2021/04/19/press-briefing-by-white-house-covid-19-response-team-and-public-health-officials-30/

